# Subclinical cardiac perforation by cardiac implantable electronic device leads detected by cardiac computed tomography

**DOI:** 10.1186/s12872-021-02159-3

**Published:** 2021-07-19

**Authors:** Yeong-Min Lim, Jae-Sun Uhm, Min Kim, In-Soo Kim, Moo-Nyun Jin, Hee Tae Yu, Tae-Hoon Kim, Hye-Jeong Lee, Young-Jin Kim, Boyoung Joung, Hui-Nam Pak, Moon-Hyoung Lee

**Affiliations:** 1 Division of Cardiology, Department of Internal Medicine, Saint Carollo Hospital, Suncheon, Jeollanam-do Republic of Korea; 2 Department of Cardiology, Yongin Severance Hospital, Yonsei University College of Medicine, 363 Dongbaekjukjeondaero Giheung-gu Yongin, Seoul, Gyeonggi-do Republic of Korea; 3 Department of Radiology, Severance Hospital, Yonsei University College of Medicine, Seoul, Republic of Korea

**Keywords:** Cardiac computed tomography, Cardiac implantable electronic device, Cardiac perforation, Complications

## Abstract

**Background:**

The relationship between the characteristics of cardiac implantable electronic device (CIED) leads and subclinical cardiac perforations remains unclear. This study aimed to evaluate the incidence of subclinical cardiac perforation among various CIED leads using cardiac computed tomography (CT).

**Methods:**

A total of 271 consecutive patients with 463 CIED leads, who underwent cardiac CT after CIED implantation, were included in this retrospective observational study. Cardiac CT images were reviewed by one radiologist and two cardiologists. Subclinical perforation was defined as traversal of the lead tip past the outer myocardial layer without symptoms and signs related to cardiac perforation. We compared the subclinical cardiac perforation rates of the available lead types.

**Results:**

A total of 219, 49, and 3 patients had pacemakers, implantable cardioverter-defibrillators, and cardiac resynchronization therapy, respectively. The total subclinical cardiac perforation rate was 5.6%. Subclinical cardiac perforation by screw-in ventricular leads was significantly more frequent than that caused by tined ventricular leads (13.3% vs 3.3%, respectively, *p* = 0.002). There were no significant differences in the incidence of cardiac perforation between atrial and ventricular leads, screw-in and tined atrial leads, pacing and defibrillator ventricular leads, nor between magnetic resonance (MR)-conditional and MR-unsafe screw-in ventricular leads. Screw-in ventricular leads were significantly associated with subclinical cardiac perforation [odds ratio, 4.554; 95% confidence interval, 1.587–13.065, *p* = 0.005]. There was no case subclinical cardiac perforation by septal ventricular leads.

**Conclusions:**

Subclinical cardiac perforation by screw-in ventricular leads is not rare. Septal pacing may be helpful in avoiding cardiac perforation.

**Supplementary Information:**

The online version contains supplementary material available at 10.1186/s12872-021-02159-3.

## Introduction

Asymptomatic cardiac perforations by cardiac implantable electronic device (CIED) leads are common (6–20%) [1]. The incidence of implantations-related perforations are more than is clinically appreciated. However, some cardiac perforation may remain unnoticed because they do not result in symptoms, hemodynamic changes, or abnormalities in the functions of the device [2–5]. The clinical significance and natural history of these perforations are uncertain; however, many case reports have described instances in which delayed perforation caused chest pain, pericardial effusion, and pneumothorax. There are even reports of leads migrating to the chest wall or below the diaphragm [6–11]. These reports suggest that subclinical cardiac perforations by CIED leads may lead to clinical cardiac perforations.

Previous studies showed confusing data about the cardiac perforation rate among various leads and definite risk factors for asymptomatic cardiac perforation [1, 5, 12]. Moreover, there are concerns on cardiac perforation by first-generation magnetic resonance (MR)-conditional leads [13–15]. Cardiac computed tomography (CT) with multiplane reformatting is useful for documenting lead position and assessing possible cardiac perforation [16]. This study aimed to compare the incidence of subclinical cardiac perforation among various CIED leads, and evaluate the anatomical distribution of CIED lead-related cardiac perforations using cardiac CT.

## Methods

### Study population

This is a retrospective observational study. The study design was approved by the Institutional Review Board of the Yonsei University Health System (Approval Number: 4–2019-0661) and was conducted in accordance with the guidelines stipulated by the Declaration of Helsinki. The institutional review board waived both the need for the acquisition of informed consent from the patients to be included in the analysis and the need for review by a critical event committee because of the study’s retrospective nature and the absence of data that could be used to identify patients in this study.

Patients who underwent cardiac CT after CIED implantation in a tertiary hospital were retrospectively included in this study. The inclusion criteria were as follows: (1) patients > 18 years; (2) patients with CIED (pacemaker, implantable cardioverter-defibrillator (ICD), and cardiac resynchronization therapy (CRT)), who underwent cardiac CT for cardiovascular anatomy or coronary artery disease assessment after CIED implantation; and 3) patients with CIED interrogation data (pacing threshold, P or R wave amplitude, and impedance). The exclusion criteria were as follows: 1) epicardial CIED leads; 2) poor quality of CT images (e.g., presence of severe metallic artifacts); 3) absence of short-axis CT image; and 4) patients with symptoms and signs that were suggestive of cardiac perforation (such as, pleuritic chest pain, dyspnea, pericardia effusion, pleural effusion, and pneumothorax). Cardiac CT images of 463 CIED leads in 271 consecutive patients from February 2006 to May 2019, were reviewed by one radiologist and two cardiologists (Additional file 1: Figure S1).

### CIED implantation technique

For all right ventricular (RV) “septal” implant cases, the standard practice is to target the middle RV septum using a hand-fashioned stylet with a proximal large primary curve and a smaller distal secondary posterior curve as described by Rosso et al. [17] RV apical leads are then implanted with a slightly curved or straight stylet. In all participants in this study, right atrial (RA) leads were implanted in the standard manner with a curved J-shaped stylet. After conventional implantation of RV and RA leads with passive or active fixation, left ventricle (LV) pacing lead implantation is usually performed via a transvenous approach, which cannulates one of the tributaries of the coronary sinus.

### Cardiac CT

For each patient, cardiac CT was performed using multidetector CT systems (Aquilion ONE; Toshiba Medical Systems, Tokyo, Japan) or Light Speed Volume CT scanners (Philips, Brilliance 63, Amsterdam, The Netherlands). Cardiac phase reconstruction images were taken, usually at the mid diastole which corresponds to approximately 70%-80% of the RR interval. A slice thickness of 0.75 mm was used, with incremental interval of 0.5 mm in axial source data of cardiac CT. Using an image reconstructed in mid-diastole, orthogonal oblique multiplanar reformats were created with slice thickness of 1 mm, incremental interval of 1 mm. The images of CT were analyzed mainly in a mediastinal setting using a center around 50 Hounsfield unit (HU) and a narrower width of approximately 400 HU.

### Definition of terms

MR-conditional leads were defined as CIED leads that were initially designed for MR scanning (e.g., CapSureFix MRI 5086 lead, Medtronic; Tendril MRI lead, Abbott; and Ingevity MRI lead, Boston Scientific). MR-unsafe leads were defined as CIED leads that were not initially designed for MR scanning. Subclinical perforation was defined as traversal of the lead tip past the outer myocardial layer from at least two different views in cardiac CT, without symptoms and signs related to cardiac perforation [16].

### Anatomical distributions of RV lead in patients with CIED

**Fig. 1:**
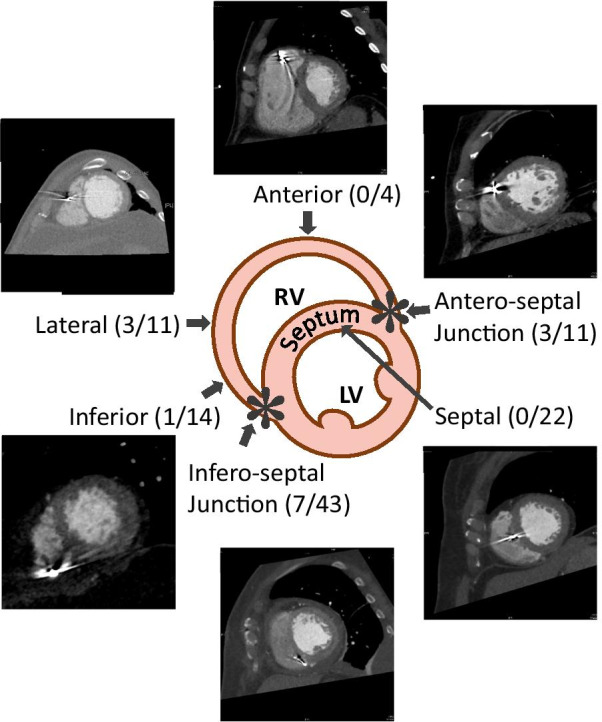
Anatomical distribution of subclinical perforation by screw-in right ventricular lead in the short axis view of cardiac CT

RV lead positions were categorized according to the short-axis views of cardiac CT. We hypothesized that positioning the lead in the thicker, non-apex site instead of the traditional RV apex may alleviate the risks of cardiac perforation. In the short-axis views of the RV, we established 6 anatomical categories of leads [16]. The actual ventricular lead locations observed were in the infero-septal junction, inferior, lateral, anterior, antero-septal junction, and septal locations (Fig. 1). In the long-axis views of cardiac CT of the RV, the lead positions were divided into 3 anatomical categories: namely, RV outflow tract, middle RV, and RV apex. In the chest posteroanterior (PA) view, the region from the pulmonary artery bulge to the inferior border of the cardiac silhouette was divided into three equal parts by horizontal lines, similar to what was done in a previous study [18]. The inferior third on cardiac CT scans and PA view on chest X-ray films was defined as the RV apex.

### Statistical analysis

Continuous variables are reported as medians (interquartile range) and were analyzed using the Wilcoxon rank sum test and Mann–Whitney U test. Categorical variables were reported as counts and proportions and analyzed using Pearson chi-square test or Fisher’s exact test as appropriate. A logistic regression analysis was conducted to determine odds ratios (OR) for subclinical cardiac perforation. A *P* value < 0.05 was considered to be statistically significant. Statistical analyses were performed using SPSS version 23.0 (IBM Corp., Armonk, NY, USA).

## Results

### Study population

A total of 271 patients [age 70.5 (60.0–78.1) years; 49.8% males], comprising 219 patients with permanent pacemaker, 49 with ICD, and 3 patients receiving CRT, were evaluated in this study. There were 128 patients (47.2%) with structural heart disease, such as heart failure with reduced ejection fraction (11.8%), coronary artery disease (15.9%), hypertrophic cardiomyopathy (3.3%), severe valvular disease (21.0%), and congenital heart disease (6.3%). CT scans were used to evaluate the cardiovascular anatomy (pulmonary vein for ablation, aorta for intervention and post-operative evaluation after coronary artery bypass surgery) (189/271, 69.7%) or to evaluate co-existing coronary artery disease in structural heart disease patients (82/271, 30.2%). The radiation dose of CT was calculated using a dose-length product, and the mean value was 483.6 ± 399.4 mGycm for cardiac CT. Table 1 shows the baseline characteristics of the study population. There were no significant differences in the baseline characteristics between patients with subclinical perforations and those without subclinical perforations. The time interval from CIED implantation to CT scanning was 1.6 (0.3–4.1) years.

**Table 1:** Baseline characteristics of study population

	Patients with subclinical perforation (n = 25)	Patients without subclinical perforation (n = 246)	*p*
Age (year)	62.2 (53.8–77.8)	71.7 (60.8–78.3)	0.174
Male	12 (48.0)	123 (50.0)	0.849
Pacemaker	19 (76.0)	200 (81.3)	0.593
DDD	17 (68.0)	137 (55.7)^*^	0.222
DDD with LV lead	0 (0.0)	3 (1.2)	–^†^
VVI	2 (8.0)	46 (18.7)^‡^	0.617
AAI	0 (0.0)	14 (5.7)^§^	–^†^
ICD	6 (24.0)	43 (17.4)	0.417
Single-chamber	3 (12.0)^¶^	22 (8.9)	0.999
Dual-chamber	3 (12.0)	21 (8.5)	0.473
CRT-D	0 (0.0)	3 (1.2)	–^†^

Values are presented as median (interquartile range) or as n (%)

*CRT-D* cardiac resynchronization therapy with defibrillator, *ICD* implantable cardioverter-defibrillator, *LV* left ventricle, *RA* right atrium, *RV* right ventricle

^*^One patient with DDD pacemaker with 2 RV leads was included

^†^Statistical comparison could not be performed because the number of patients was small

^‡^Two patients with VVI pacemaker with 2 RV leads were included

^§^One patient with AAI pacemaker with 2 atrial leads was included

^¶^One patient with ICD with 2 defibrillation leads was included

### Subclinical cardiac perforation

**Fig. 2:**
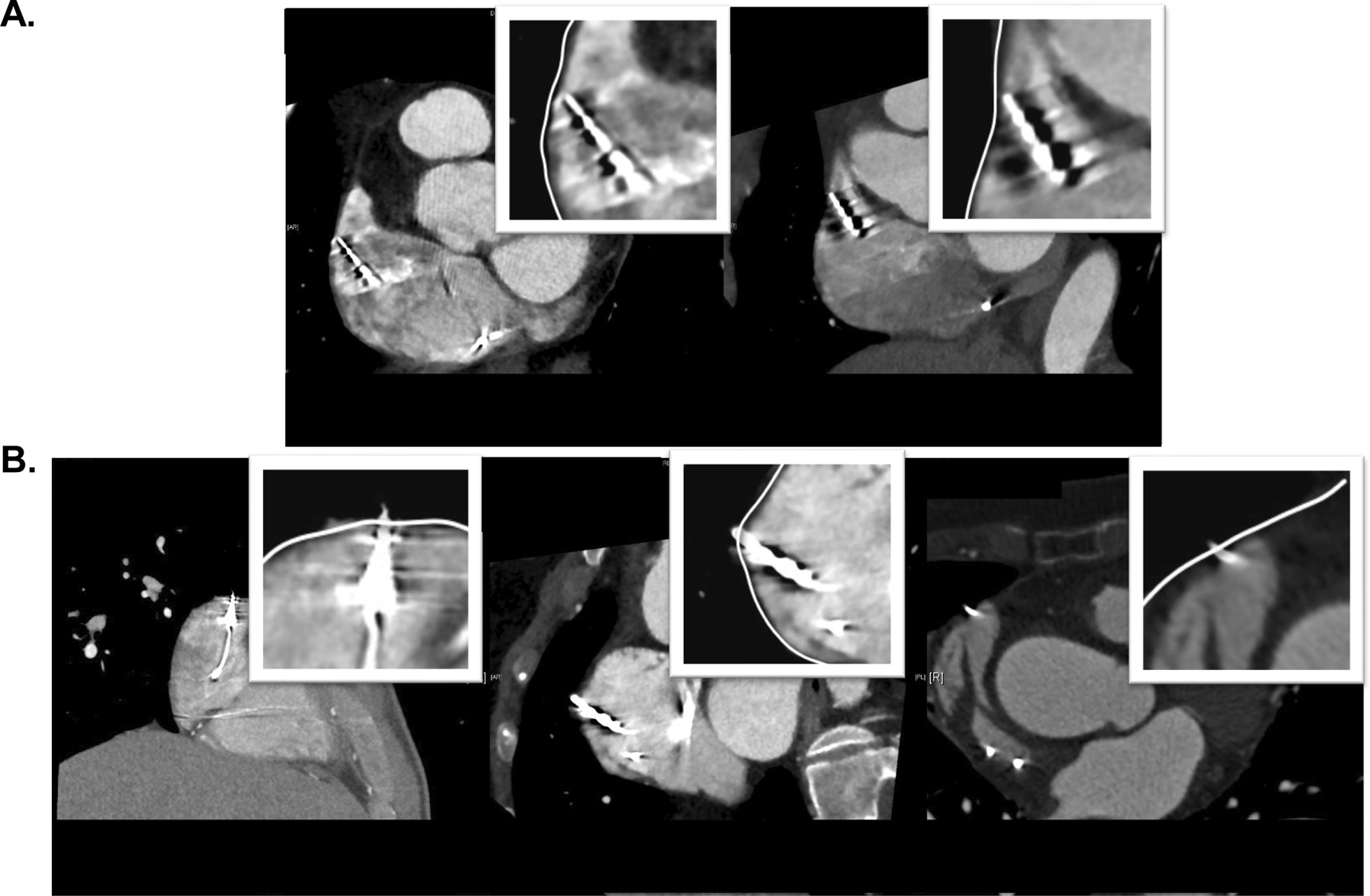
The examples of nonperforated (**a**) and subclinical perforated (**b**) CIED leads in the right atrium. In Fig. 2B, the atrial leads have protruded from the outer edge of the myocardium (white line). CIED, cardiac implantable electronic device

**Fig. 3:**
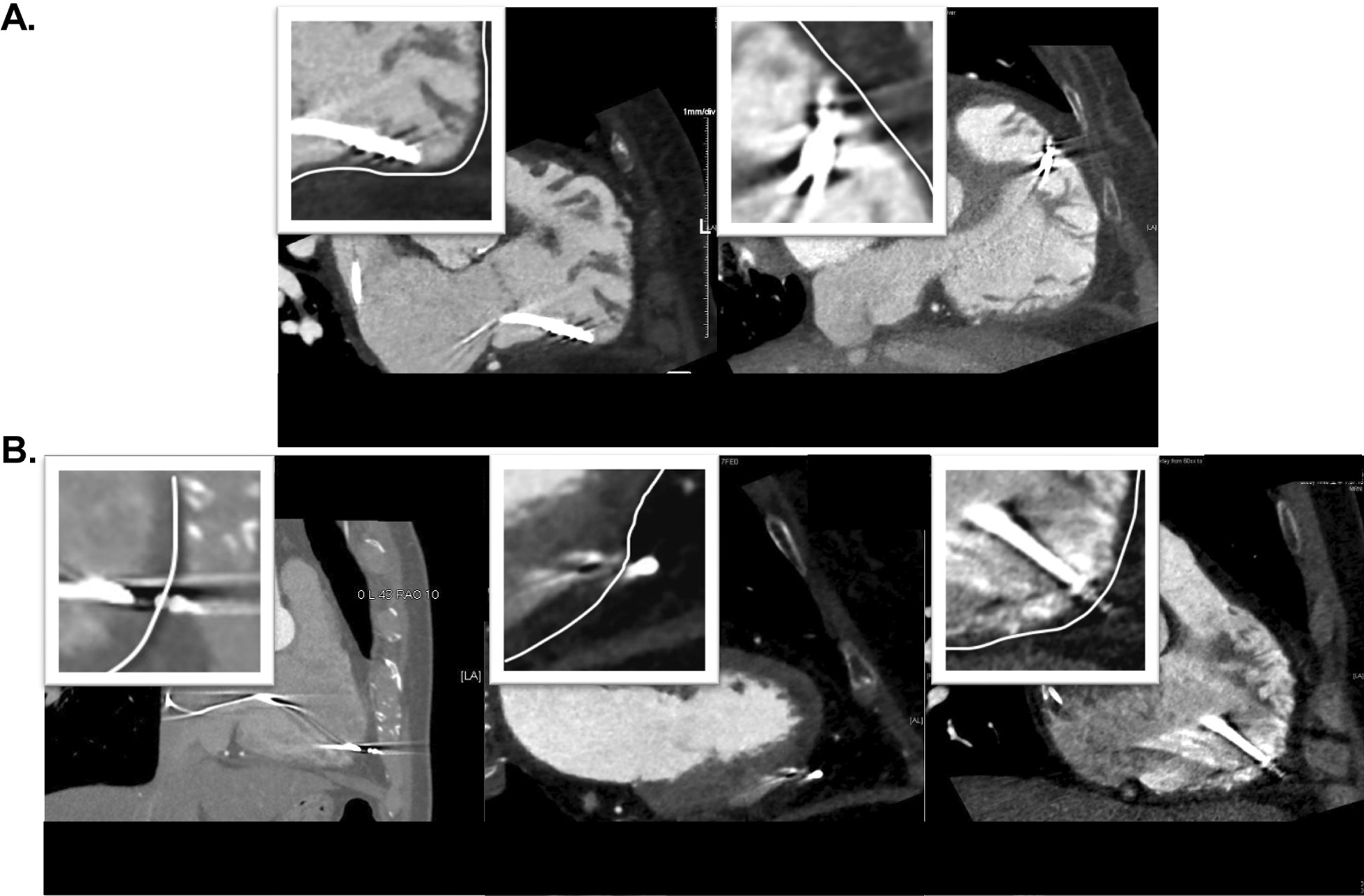
The examples of nonperforated (**a**) and subclinical perforated (**b**) CIED leads in the right ventricle. In Fig. 3B, the ventricular leads have protruded from the outer edge of the myocardium (white line). CIED, cardiac implantable electronic device

The subclinical cardiac perforation rate of all leads was 5.6% (26/463). Figure 2 shows the examples of nonperforated (A) and subclinical perforated (B) CIED leads in the right atrium. Figure 3 demonstrates the examples of nonperforated (A) and subclinical perforated (B) CIED leads in the right ventricle. Subclinical cardiac perforation by screw-in ventricular leads was significantly more frequent than that by tined ventricular leads (13.3% vs 3.3%, *p* = 0.002). There were no significant differences in the incidence of cardiac perforation between atrial and ventricular leads (3.5% vs 7.4%, *p* = 0.078), screw-in and tined atrial leads (3.8% vs 3.2%, *p* = 0.999), pacing and defibrillator ventricular leads (6.3% vs 11.3%, *p* = 0.238), nor between MR-conditional and MR-unsafe screw-in ventricular leads (12.2% vs 14.3%, *p* = 0.765) (Table 2). In the logistic regression analysis, screw-in ventricular leads were significantly associated with subclinical cardiac perforations [OR, 4.554; 95% confidence interval, 1.587–13.065, *p* = 0.005] (Table 3). However, age, sex, structural heart disease, atrial/ventricular leads, screw-in/tined atrial leads, pacing/defibrillation lead, MR-conditional lead, and RV apical leads were not associated with subclinical cardiac perforations. There were no significant differences in the R-wave sensing, stimulation thresholds, or measured impedances at the time of implantation and after the cardiac CT scan except one atrial sensing failure and one atrial increased pacing threshold not associated with subclinical perforation. There was no development of clinical cardiac perforations among patients with subclinical cardiac perforations for 2.7 (1.1–4.5) years.

**Table 2:** The prevalence of subclinical cardiac perforation by various leads

	Subclinical perforation rate	*p*
Atrial lead	7/199(3.5)	
Fixation type		0.999
Tined	3/93 (3.2)	
Screw-in	4/106 (3.8)	
MR condition^*^		–^†^
MR-conditional	0/39 (0.0)	
MR-unsafe	4/50 (8.0)	
RV lead	19/258 (7.4)	
Lead type		0.238
Pacing	13/205 (6.3)	
Defibrillator	6/53 (11.3)	
Fixation type		0.002
Tined	5/153 (3.3)	
Screw-in	14/105 (13.3)	
MR condition^‡^		0.765
MR-conditional	5/41 (12.2)	
MR-unsafe	8/56 (14.3)	
RV lead position-1		0.761
Apex	15/210 (7.1)	
Non-apex	4/48 (8.3)	
RV lead position-2		–^†^
Septum	0/56 (0.0)	
Non-septum	19/202 (9.4)	
LV lead	0/6 (0.0)	

Values are presented as n/total leads (%)

*LV* left ventricle, *MR* magnetic resonance, *RV* right ventricle

^*^Tined leads were not included. Lead profiles of 17 screw-in leads were not available

^†^Statistical comparison could not be performed because the number of patients was small

^‡^Tined leads were not included. Lead profiles of 8 screw-in leads were not available

**Table 3:** Risk for subclinical cardiac perforation by CIEDs

	OR (95% CI)	*p*
Age (per year)	0.985 (0.960–1.010)	0.228
Male sex	0.923 (0.405–2.103)	0.849
Structural heart disease	0.866 (0.378–1.984)	0.734
Ventricular lead	2.181 (0.898–5.295)	0.085
Screw-in atrial lead	1.176 (0.256–5.398)	0.834
Screw-in ventricular lead	4.554 (1.587–13.065)	0.005
Defibrillation lead	1.885 (0.681–5.221)	0.222
MR-conditional lead	0.854 (0.307–2.376)	0.763
RV apex lead	0.846 (0.268–2.673)	0.776

*CIED* cardiac implantable electronic device, *CI* confidence interval, *MR* magnetic resonance, *OR* odds ratio, *RV* right ventricle

### Anatomical distributions of subclinical CIED screw-in lead-related perforations viewed by cardiac CT

Figure 1 shows the anatomical distributions of CIED screw-in leads in the short-axis views of cardiac CT. In the six anatomical categories of the RV lead position, the subclinical cardiac perforation rates of screw-in lead were: infero-septal junction (7/43, 16.3%), inferior (1/14, 7.1%), lateral (3/11, 27.3%), anterior (0/4, 0%), antero-septal junction (3/11, 27.3%), and septal (0/22, 0%). There was no subclinical cardiac perforation by septal screw-in ventricular leads.

## Discussion

### Main findings

The main findings of this study are: (1) the subclinical cardiac perforation rate by CIED leads was 5.6%; (2) the rate of subclinical cardiac perforation by screw-in ventricular leads was higher than that by tined ventricular leads; and (3) there was no subclinical cardiac perforation by septal ventricular leads.

Clinical perforation was reported as < 1% [7]. Patients with cardiac perforation by CIED leads may exhibit no or minimal symptoms and remain undetected in clinical practice if hemopericardium does not develop or the perforating CIED leads do not irritate the pericardium or pleura. This may be because the incidence of cardiac perforation by CIED was higher in this study than in clinical practice.

### Increased perforation risk of screw-in ventricular leads

Given that screw-in leads provide secure fixation of the leads, they have played a dominant role in current CIED lead implantation procedures. If screw-in leads are screwed excessively, screw-in leads with a 2-mm helical screw may penetrate the myocardium by tunneling into the RV wall with a 4–5 mm thickness. In addition, a fixed straight lead may transfer more vector forces to the underlying myocardium than curved leads [16]. This hypothesis can explain the reason why that the frequency of subclinical perforation in tined leads was comparable with that in screw-in leads in atrium in our study. Because all RA leads are curved leads, it seems to that screwing and over-torquing had less impact on atrial myocardium under low vector force. Recent two study’s data were consistent with our study findings [16, 19]. Contrary to our results, one study showed that the incidence of clinically relevant cardiac perforation associated with implantation of active-fixation leads is low and comparable to that reported with the use of passive-fixation leads [5]. It cannot be overemphasized that one must not screw the CIED leads excessively in order to avoid cardiac perforations.

### Delayed cardiac perforation by CIED leads

Generally, delayed perforation was defined as a cardiac perforation occurring > 1 month after CIED implantation [7]. As it was difficult to determine the exact time of perforation, it was unclear whether the subclinical cardiac perforations observed in the patients who had implants for > 1 month were delayed or not. Delayed cardiac perforation by CIED leads is an under-recognized complication with significant morbidity and mortality, particularly if not detected early [11]. The pathophysiology of delayed cardiac perforation is not clearly understood. Delayed cardiac perforation might result from increased pressure exerted by the thin CIED leads per unit area of ventricular wall, as well as imbalance between the force exerted by the tips of the CIED leads and the ventricle.

### Septal pacing strategy for avoiding cardiac perforation

The question of whether RV septal pacing is associated with better clinical outcomes compared to RV apical pacing, remains controversial due to the lack of large-scale randomized controlled studies. Positioning the lead in the thicker non-apical wall instead of the traditional site at the RV apex is thought to mitigate the risk of cardiac perforation [4, 20, 21]. Pacing leads may be subjected to more motions at the RV apex than at the RV non-apical sites. In some recent studies, compared with RV apical pacing, septal pacing was associated with lower mortality and had fewer adverse effects in terms of atrial electrical activity and structure [22, 23]. RV septal pacing may be beneficial because it poses a lesser risk of cardiac perforation compared to other leads.

### Study limitations

This was a single-center retrospective observational study. Given that the patients with CIED who did not undergo cardiac CT were not included in the present study, a potential selection bias might have occurred. Although patients with pleuritic chest pain were excluded, angina, which was the reason why cardiac CT was performed, might have been a symptom associated with cardiac perforation by the CIED leads. Beam hardening, bloom, and motion artifacts of the high-density metallic pacing leads remain issues inherent in any CT study. The artifacts at the lead tip could make the assessment of subtle perforations difficult. For CRT, only few patients were enrolled, which made it difficult to assess subclinical perforation of LV leads. Finally, we did not use direct measurement by thoracoscopy or other means such as right ventriculography to confirm subclinical cardiac perforation by CIED leads.

## Conclusions

Subclinical cardiac perforation by screw-in ventricular CIED leads is not rare. Septal pacing may be helpful in reducing the risk of cardiac perforations by screw-in ventricular CIED leads.

## Supplementary Information

**Additional file 1.** Study population.

## Data Availability

The dataset analyzed in the current study is not publicly available due to lack of consent from study participants to do so but it is available from the corresponding author on reasonable request for researchers who meet the criteria for access to confidential data.

## Abbreviations

CIED: Cardiac implantable electronic device
CT: Computed tomography
MR: Magnetic resonance
ICD: Implantable cardioverter-defibrillator
CRT: Cardiac resynchronization therapy
RV: Right ventricle
RA: Right atrium
LV: Left ventricle
HU: Hounsfield unit
PA: Posteroanterior
OR: Odds ratio
