# Supplementary material for: Subclinical cardiac perforation by cardiac implantable electronic device leads detected by cardiac computed tomography
Source: BMC Cardiovasc Disord. 2021 Jul 19;21:346. doi: 10.1186/s12872-021-02159-3 (PMC8290553; doi:10.1186/s12872-021-02159-3)
Supplement: Supplementary file 1 — Additional file 1. Study population. [file 12872_2021_2159_MOESM1_ESM.docx]

**Supplemental materials**

**Supplemental Figure 1.** Study population

445 were excluded owing to reason as follows:

1) epicardial CIED leads

2) poor quality of CT images (e.g. presence of severe metallic artifacts)

3) absence of short-axis CT image

4) patients with symptoms and signs that were suggestive of cardiac perforation (including pleuritic chest pain, dyspnea, pericardia effusion, pleural effusion, pneumothorax, CIED lead malfunction, and so on)

716 who underwent cardiac CT after CIED implantation with interrogation data (pacing threshold, P or R wave amplitude, and impedance) between 2006~2019 were assessed for eligibility

271 constituted the final study population

CIED, cardiac implantable electronic device; CT, computed tomogram
